# Carcinoma-Associated Mesenchymal Stem Cells Promote Chemoresistance in Ovarian Cancer Stem Cells via PDGF Signaling

**DOI:** 10.3390/cancers12082063

**Published:** 2020-07-27

**Authors:** Shreya Raghavan, Catherine S. Snyder, Anni Wang, Karen McLean, Dmitriy Zamarin, Ronald J. Buckanovich, Geeta Mehta

**Affiliations:** 1 Department of Materials Science and Engineering, University of Michigan, Ann Arbor, MI 48109, USA; shreyar@umich.edu (S.R.); cssnyder@umich.edu (C.S.S.); 2 Department of Biomedical Engineering, University of Michigan, Ann Arbor, MI 48109, USA; anniwang@umich.edu; 3 Department of Obstetrics and Gynecology, Division of Gynecologic Oncology, Michigan Medicine, University of Michigan, Ann Arbor, MI 48109, USA; karenmcl@umich.edu; 4 Rogel Cancer Center, University of Michigan, Ann Arbor, MI 48109, USA; 5 Department of Gynecologic Medical Oncology and Immunotherapeutics, Memorial Sloan Kettering Cancer Center, New York, NY 10065, USA; zamarind@mskcc.org; 6 Director of Ovarian Cancer Research, Magee Womens Research Institute, University of Pittsburgh, Pittsburgh, PA 15260, USA; buckanovichrj@mwri.magee.edu; 7 Department of Macromolecular Sciences and Engineering, University of Michigan, Ann Arbor, MI 48109, USA; 8 Precision Health, University of Michigan, Ann Arbor, MI 48109, USA

**Keywords:** ovarian cancer, cancer stem-like cells (CSC), carcinoma associated mesenchymal stem cells (CA-MSC), platelet derived growth factor (PDGF), stemness, chemoresistance, high grade serous ovarian cancers, 3D spheroids, tumoroids, heterospheroids, stromal cells

## Abstract

Within the ovarian cancer tumor microenvironment, cancer stem-like cells (CSC) interact with carcinoma associated mesenchymal stem/stromal cells (CA-MSC) through multiple secreted cytokines and growth factors. These paracrine interactions have been revealed to cause enrichment of CSC and their chemoprotection; however, it is still not known if platelet-derived growth factor (PDGF) signaling is involved in facilitating these responses. In order to probe this undiscovered bidirectional communication, we created a model of ovarian malignant ascites in the three-dimensional (3D) hanging drop heterospheroid array, with CSC and CA-MSC. We hypothesized that PDGF secretion by CA-MSC increases self-renewal, migration, epithelial to mesenchymal transition (EMT) and chemoresistance in ovarian CSC. Our results indicate that PDGF signaling in the CSC-MSC heterospheroids significantly increased stemness, metastatic potential and chemoresistance of CSC. Knockdown of *PDGFB* in MSC resulted in abrogation of these phenotypes in the heterospheroids. Our studies also reveal a cross-talk between PDGF and Hedgehog signaling in ovarian cancer. Overall, our data suggest that when the stromal signaling via PDGF to ovarian CSC is blocked in addition to chemotherapy pressure, the tumor cells are significantly more sensitive to chemotherapy. Our results emphasize the importance of disrupting the signals from the microenvironment to the tumor cells, in order to improve response rates. These findings may lead to the development of combination therapies targeting stromal signaling (such as PDGF and Hedgehog) that can abrogate the tumorigenic, metastatic and platinum resistant phenotypes of ovarian CSC through additional investigations.

## 1. Introduction

Ovarian cancer is the fifth leading cause of cancer-related deaths in women in the United States, and has a low survival rate, due in part to the frequent late stage at diagnosis [1,2]. Despite aggressive surgical debulking and first-line platinum-based therapy, a ~70% relapse rate is observed, attributable to chemoresistance and peritoneal metastasis [1]. Chemoresistance and new tumor growth following primary therapy has been ascribed to ovarian cancer stem cells (CSC), through a complex interplay with other cells within the tumor microenvironment (TME) [1,3,4,5,6,7]. Previous studies have indicated that CSC in ovarian cancer are identified with high CD133 expression and increased aldehyde dehydrogenases (ALDH) activity, and presence of these cells predicts prognosis for reduced progression-free survival and poor patient outcome [8,9,10,11,12,13,14,15,16,17,18,19,20,21]. Additionally, elevated ALDH activity alone has been utilized in previous studies as a marker of the CSC progenies [8,9,10,11,12,13,14,15,16,17,18,19,20,21].

Stromal cells within the TME, including carcinoma-associated mesenchymal stem/stromal cells (CA-MSC) play critical supportive roles aiding in CSC proliferation, metastasis and chemoresistance [22,23,24,25]. CA-MSC arise from tumor mediated reprograming of local tissue MSC, and have been linked to increased tumorigenesis and chemoresistance through secretion of a complex cytokine network involving IL-6, SDF-1, TGF-β, BMP2, BMP4 and CCL5 [22,23,26,27,28]. Co-culture of ovarian cancer cells with MSC leads to increased proliferation, invasiveness and platinum resistance of the cancer cells [29,30]. Additionally, ovarian CSC and CA-MSC are known to communicate bi-directionally, with CSCs possessing the ability to impact the phenotype of MSCs to make them more tumor-supportive [26]. Within the framework of this dynamic CSC/CA-MSC relationship, we hypothesize that platelet-derived growth factor (PDGF) plays a critical role in CSC/CA-MSC pro-tumorigenic signaling.

The PDGF family has been shown to play a crucial role in MSC differentiation and growth [31] and can protect MSC from apoptosis and senescence [32]. Additionally, in ovarian cancers, PDGF ligands, including PDGF-BB, and their corresponding receptors (PDGFR) have been noted as a potential key pathway with poor prognosis, yet PDGF signaling is poorly studied in the context of ovarian CSC/CA-MSC interactions [33,34,35,36]. PDGFR are known to promote CSC phenotypes in sarcomas [37]. PDGF signaling has also been linked to the formation and maintenance of glioma, gastric cancer, lung cancer and breast cancer, among others [38,39,40,41,42,43]. Downstream of the PDGF-BB/PDGFR interaction, several mechanistic pathways have been shown to increase tumorigenicity, metastasis and CSC phenotypes. Among those documented are the epithelial to mesenchymal transition (EMT) pathway in several cancers, as well as the Hedgehog (HH) signaling pathway [44,45,46,47]. Importantly, the multitargeted tyrosine kinase inhibitor, sunitinib and Hedgehog signaling pathway (Smoothened) inhibitor, sonidegib, have been explored in randomized phase II settings, demonstrating moderate single agent efficacy in targeting stromal components in ovarian and other cancers [48,49,50,51,52,53,54].

In this study, we utilized a three-dimensional (3D) hanging drop array spheroid platform, to bring CSC and human adipose MSC (MSC) or carcinoma-associated primary MSC (CA-MSC) in close association in a non-adherent 3D in vitro structure, similar to their presence and interactions within the malignant ascites [19,50,55,56,57,58]. Using this model, we aimed to investigate the hypothesized PDGF-BB/PDGFR-β signaling and its downstream effects on functional CSC behaviors, including migratory potential, tumorigenicity and platinum resistance. In addition, we evaluated the role of PDGF-BB/PDGFR-β interactions in promoting EMT and/or Hedgehog signaling, and their effect on the metastatic and tumorigenic CSC phenotype. Overall, coupled with the importance of PDGF as a prognostic marker for ovarian cancer, we believe that disrupting the trophic signaling between CA-MSC and CSC by inhibiting PDGF signaling is a novel therapeutic avenue of investigation to reduce platinum resistance in ovarian cancers.

## 2. Results

### 2.1. Characterization of CSC/MSC Heterospheroids

**Figure 1:**
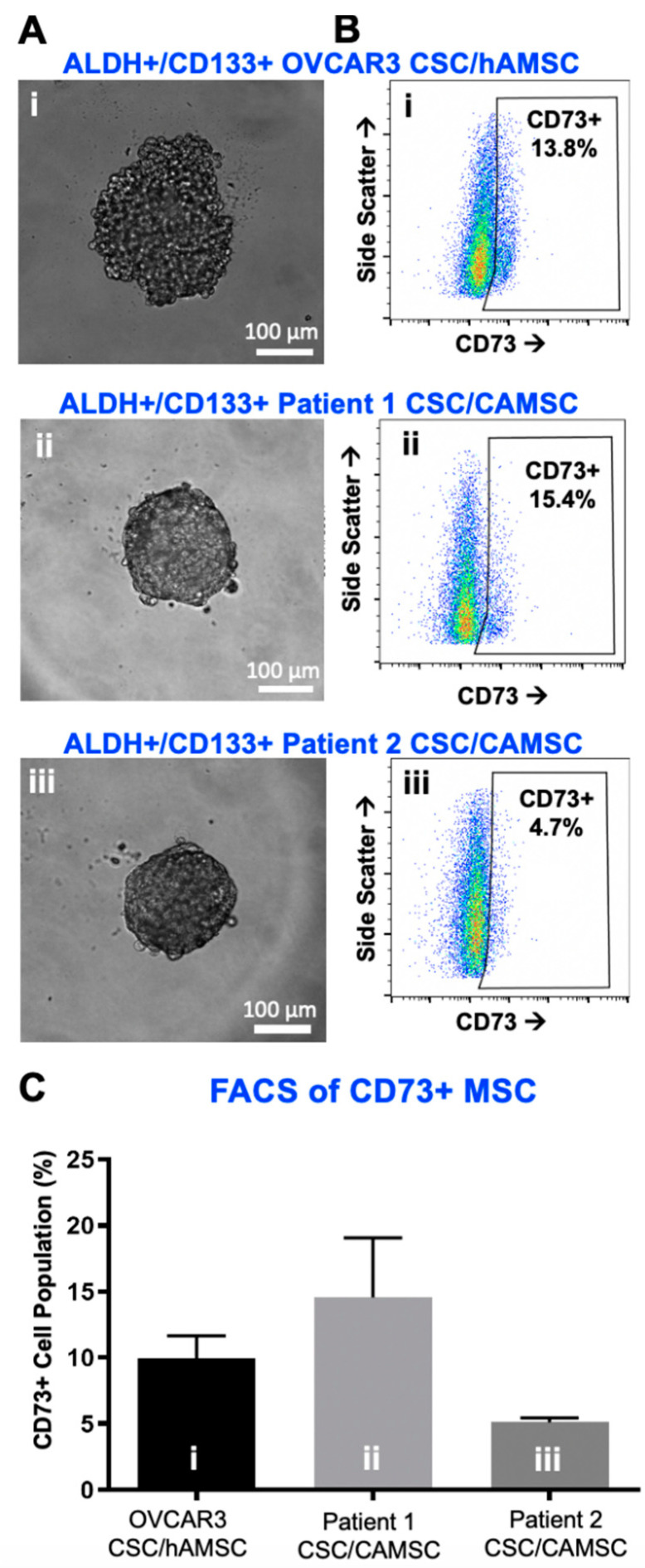
Characterization of ALDH+/CD133+ cancer stem-like cells (CSC)/carcinoma associated mesenchymal stem cell (CA-MSC) heterospheroids with fluorescence activated cell sorting (FACS). (**A**) Phase contrast micrographs of (i) OVCAR3 CSC/MSC, (ii) Patient 1 CSC/CA-MSC and (iii) Patient 2 CSC/CA-MSC heterospheroids showing compact spheroids following 4 days in hanging drop culture. (**B**) Representative flow cytometry plots quantifying the presence of CD73+ MSC within heterospheroids (i) OVCAR3 CSC/MSC, (ii) Patient 1 CSC/CA-MSC and (iii) Patient 2 CSC/CA-MSC heterospheroids at Day 5. See Supplementary Figures S1–S4 for gating strategies. (**C**) Graphical representation of flow cytometry analysis data, quantifying the presence of CD73+ MSC within heterospheroids, ranging from ~5% to 27% (*n* ≥ 3).

CSC/MSC heterospheroids (either human ovarian carcinoma OVCAR3/MSC or matched patient CSC/CA-MSC) were generated and observed to form compact, intact spheroids in hanging drop cultures by 4 days (Figure 1A). At Day 5, heterospheroids were harvested to characterize the presence of MSC within them using CD73. Flow analysis protocols were adapted from our previous studies [19,20,21,55,56], and gating strategy for each antibody and its associated FMO isotype are demonstrated in Figures S1–S4. Representative flow analysis plots (Figure 1B) indicate the presence of a CD73+ population within heterospheroids. Further quantification indicated the robust presence of a 5–20% CD73+ MSC population within heterospheroids at Day 5 (Figure 1C). As seen in Figure S1, CSC did not express CD73.

### 2.2. CSC/MSC Heterospheroids Demonstrate a Platinum-Resistant Phenotype, Enriched with ALDH+ Cells

**Figure 2:**
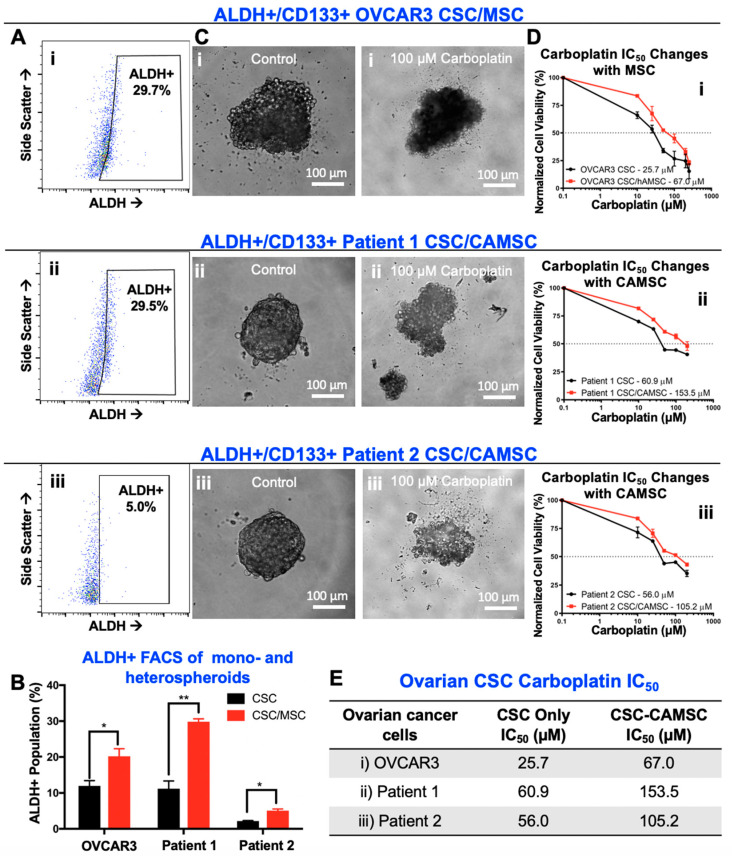
Co-culture of ovarian CSC with MSC leads to increased stemness and platinum resistance. (**A**) Representative flow cytometry plots of ALDH+ cells in (i) OVCAR3 CSC/MSC, (ii) Patient 1 CSC/CA-MSC and (iii) Patient 2 CSC/CA-MSC heterospheroids. (**B**) Graphical representation of ALDH+ flow cytometry analysis between CSC monospheroids, and CSC/MSC heterospheroids indicate a significant increase in ALDH+ populations in heterospheroids (* *p* < 0.05, ** *p* < 0.001, two-way ANOVA, *n* ≥ 3). (**C**) Phase contrast micrographs of (i) OVCAR3 CSC/MSC, (ii) Patient 1 CSC/CA-MSC and (iii) Patient 2 CSC/CA-MSC Day 5 heterospheroids treated with 100 µM of carboplatin or control untreated for 48 h. Images indicate a loss of boundary integrity and cell death within carboplatin treated spheroids, compared to untreated spheroids at the same time-point. (**D**) Graphical representation of IC_50_ plots derived from cell viability quantification, demonstrating that CSC/MSC heterospheroids demonstrate an increased IC_50_ to carboplatin compared to CSC monospheroids (*n* ≥ 3). (**E**) Tabulation of changes in IC_50_ values with MSC/CA-MSC co-culture.

To assess the impact of MSC on cancer cell stemness, we evaluated aldehyde dehydrogenase (ALDH) activity within heterospheroids as a marker of CSC, consistent with our prior work [28]. Previous work has established ovarian cancer cells that express CD133 highly and have elevated ALDH activity, as the ALDH+ CD133+ CSC, which are at the apex of the CSC hierarchy [8,9,11,18,19,20,21,55,59]. These CSC and can self-renew and asymmetrically divide to create daughter progenies that are either ALDH+ or CD133+. Therefore, ALDH+ population contains both CD133+ and CD133− cancer cells. We found significant increase in ALDH+ cells in CSC/MSC heterospheroids when compared to CSC mono-spheroids (Figure 2A,B). Significant enhancement in ALDH+ cells (* *p* < 0.05, ** *p* < 0.001, two-way ANOVA) was observed between OVCAR3 CSC and OVCAR3 CSC/MSC spheroids, as well as Patient 1 and Patient 2 CSC/CA-MSC spheroids. On average, the ALDH+ cells increased by ~1.5-fold in CSC/MSC spheroids compared to CSC spheroids alone (Figure 2B). As seen in Figure S2C, MSC did not express ALDH.

Given the elevation in ALDH activity suggestive of increased stemness, we tested the impact of MSC on carboplatin sensitivity in CSC/MSC heterospheroids. We observed a significant elevation in carboplatin IC_50_ in CSC/MSC spheroids compared to CSC spheroids alone, in the spheroids based on OVCAR3 and primary patient samples. Representative images of control and drug treated spheroids at 100 µM carboplatin indicate the loss of boundary integrity and toxicity induced by drug treatment (Figure 2C). Carboplatin IC_50_ plot for each sample (Figure 2D) demonstrates significant changes in CSC/MSC spheroids. Similar to our previous observation [22], heterospheroids that contained MSC or CA-MSC were significantly more chemoresistant to carboplatin treatment, with higher IC_50_ values compared to CSC-only spheroids (Figure 2E). Given the heterogeneity in the patient samples, the IC_50_ values differed significantly between the heterospheroids generated with the OVCAR3 CSC and patient-derived CSC. These results are expected based on the previous characterization of patient-derived heterogeneity in the hanging drop spheroids [18,19,20,21,55].

### 2.3. Knockdown of MSC-Derived PDGFB Reveals the Critical Significance of PDGF-BB/PDGFR-β Signaling in Platinum Resistance and CSC Enrichment

**Figure 3:**
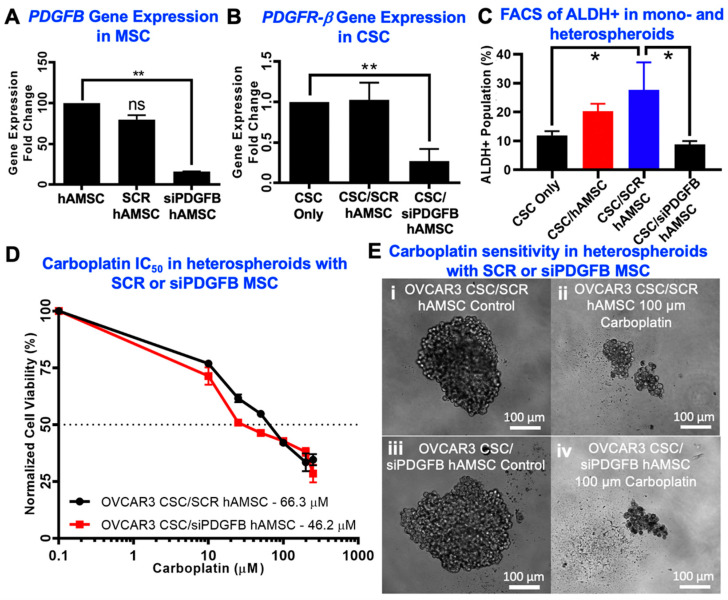
CSC platinum resistance and CSC enrichment can be reduced with *PDGFB* knockdown in MSC. (**A**) Fold change in *PDGFB* gene expression in MSCs treated with scrambled siRNA (SCR) or PDGFB siRNA (siPDGFB) was quantified with qPCR showing a significant decrease with siPDGFB (** *p* < 0.001, one-way ANOVA, *n* ≥ 3). (**B**) Gene expression of the receptor, *PDGFRB* in OVCAR3 CSCs was decreased significantly when co-cultured with siPDGFB MSCs compared to CSC mono-cultures (** *p* < 0.001, one-way ANOVA, *n* ≥ 3). (**C**) Flow cytometry of ALDH expression in OVCAR3 CSCs showed a decrease in ALDH+ cells when co-cultured with siPDGFB MSCs (* *p* < 0.05, one-way ANOVA followed by Tukey’s test, *n* ≥ 3). (**D**) Quantification of the IC_50_ changes using an MTS assay of OVCAR3 CSCs cultured with SCR or siPDGFB MSCs showing a reduction in IC_50_ (*n* ≥ 3). (**E**) Representative optical microscopy images of (i) OVCAR3 CSC/SCR MSC without drug treatment, (ii) OVCAR3 CSC/SCR MSC treated with 100 µM carboplatin, (iii) OVCAR3 CSC/siPDGFB MSC without drug treatment and (iv) OVCAR3 CSC/siPDGFB MSC treated with 100 µM carboplatin.

We hypothesized that PDGF-BB/PDGFR-β signaling may be a critical pathway involved in CSC/MSC interactions. To determine the impact of MSC PDGF signaling on platinum resistance, we proceeded to knock down *PDGFB* in MSC using siRNA. Gene expression analysis indicated that siPDGFB MSC had a 74% knockdown of *PDGFB* at 96 h, compared to no knockdown in scrambled siRNA (SCR) control MSC (** *p* < 0.001, one-way ANOVA) (Figure 3A), without significant change to viability. Heterospheroids were generated from CSC/siPDGFB MSC to understand changes in platinum resistance and percentage of ALDH+ cells upon knock-down of MSC-derived PDGF-BB. PDGFR-β gene expression was significantly reduced in CSC in CSC/siPDGFB MSC heterospheroids (** *p* < 0.001, one-way ANOVA) (Figure 3B). Increase in percent ALDH+ cells observed in SCR MSC heterospheroids was significantly reduced in CSC/siPDGFB MSC spheroids (* *p* < 0.05, one-way ANOVA; Figure 3C). Along with the significantly reduced percentage of ALDH+ cells, we also observed an increased sensitivity to carboplatin treatment, evidenced by the decreased IC_50_ values in CSC/siPDGFB MSC heterospheroids (46.2 µM compared to 66.3 µM in CSC/SCR MSC; Figure 3D). Heterospheroids generated from modified MSC were phenotypically very similar to unmodified MSC on phase contrast images (Figure 3E).

### 2.4. PDGF-BB/PDGFR-β Signaling Is Significantly Involved in CSC/MSC Interactions Leading to the Development of Platinum Resistance

**Figure 4:**
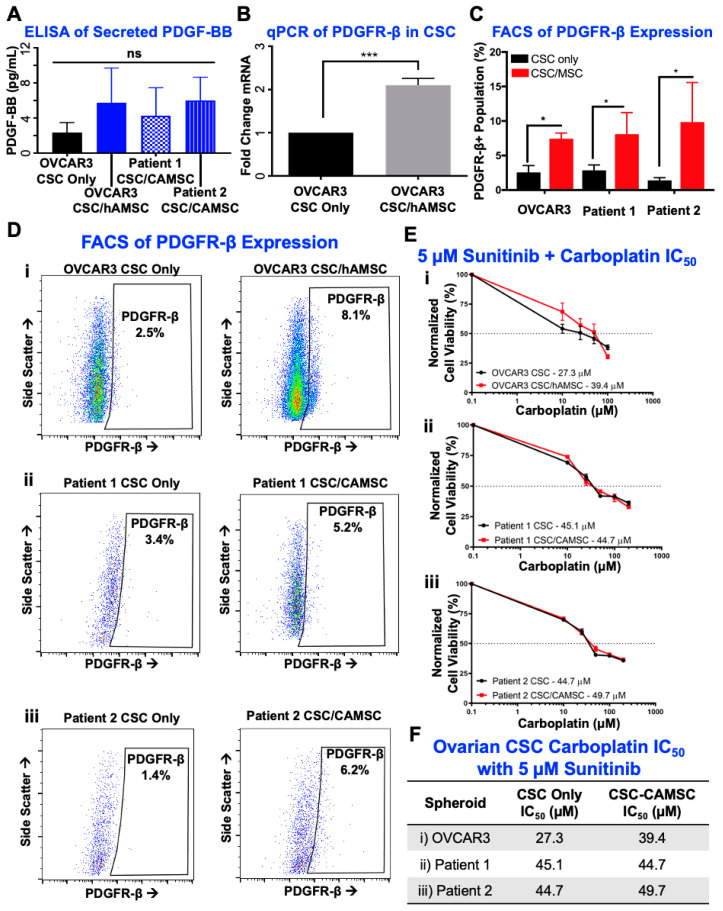
Involvement of PDGF-BB/PDGFR-β in CSC/MSC interactions leads to platinum resistance and can be inhibited with sunitinib. (**A**) Increased amounts of PDGF-BB were detected via ELISA in CSC/MSC heterospheroids, compared to CSC mono-spheroids (*n* ≥ 3). (**B**) Gene expression analysis of the receptor *PDGFRB* demonstrated a significant (*** *p* < 0.0001, *t*-test, *n* ≥ 3) >two-fold upregulation in OVCAR3 CSC co-cultured with MSCs compared to OVCAR3 CSC mono-cultures. (**C**) PDGFR-β expression was confirmed using flow cytometry, and in corroboration with qPCR data, demonstrated a non-significant increase in PDGFR-β expression in CSC/MSC heterospheroids (* *p* < 0.05, one-way ANOVA followed by Tukey’s test, *n* ≥ 3). (**D**) Representative flow cytometry plots from the quantification in (**C**). (**E**) CSC were treated with various doses of carboplatin and 5 µM of sunitinib, a PDGFR-β inhibitor, to determine the change in IC_50_ with MSC co-culture. (**F**) Tabulation of the IC_50_ values, indicating that sunitinib treatment significantly lowers carboplatin IC_50_ in CSC/CA-MSC heterospheroids.

Given that we found PDGF-BB/PDGFR-β signaling to be a critical pathway involved in CSC/MSC interactions, we next tested interactions between soluble ligand and its receptor. In order to test the presence of PDGF-BB as a soluble factor within the CSC/MSC microenvironment, we performed an enzyme-linked immunosorbent assay (ELISA), where we found that OVCAR3 CSC cultures in CSC/MSC heterospheroids demonstrated an increased presence of PDGF-BB that was not statistically significant (Figure 4A). In order to test for PDGFR-β receptor expression in CSC, we extracted RNA from OVCAR3 CSC cultured as mono-spheroids or within CSC/MSC heterospheroids. CSC from heterospheroids were sorted by flow cytometry (for ALDH activity) before RNA isolation, to avoid obtaining a signature from MSC. Our data indicate that upon MSC co-culture, CSCs significantly (*** *p* < 0.0001, unpaired *t*-test; Figure 4B) upregulate PDGFR-β expression (over two-fold).

We also analyzed PDGFR-β receptor expression using flow cytometry, and observed that CSC/MSC heterospheroids had a significantly elevated expression of PDGFR-β, compared to CSC mono-spheroids (* *p* < 0.05, one-way ANOVA with Tukey’s test; Figure 4C). Representative flow analysis plots for PDGFR-β expression are shown (Figure 4D). To inhibit the increased PDGF-BB/PDGFR-β signaling within CSC/MSC heterospheroids, we used sunitinib, which blocks PDGFR-β among other tyrosine kinases. Low dose sunitinib partially reversed MSC driven increases in chemotherapy resistance with a sub-cytotoxic dose of 5 µM, with IC_50_ 39–50 µM (Figure 4E,F) in the presence of sunitinib, compared to 67–153 µM without sunitinib (Figure 2D,E). Table S1 lists the carboplatin IC_50_ across all comparison groups, with and without sunitinib.

### 2.5. EMT Phenotype Observed Downstream of PDGF-BB/PDGFR-β Interactions in CSC

**Figure 5:**
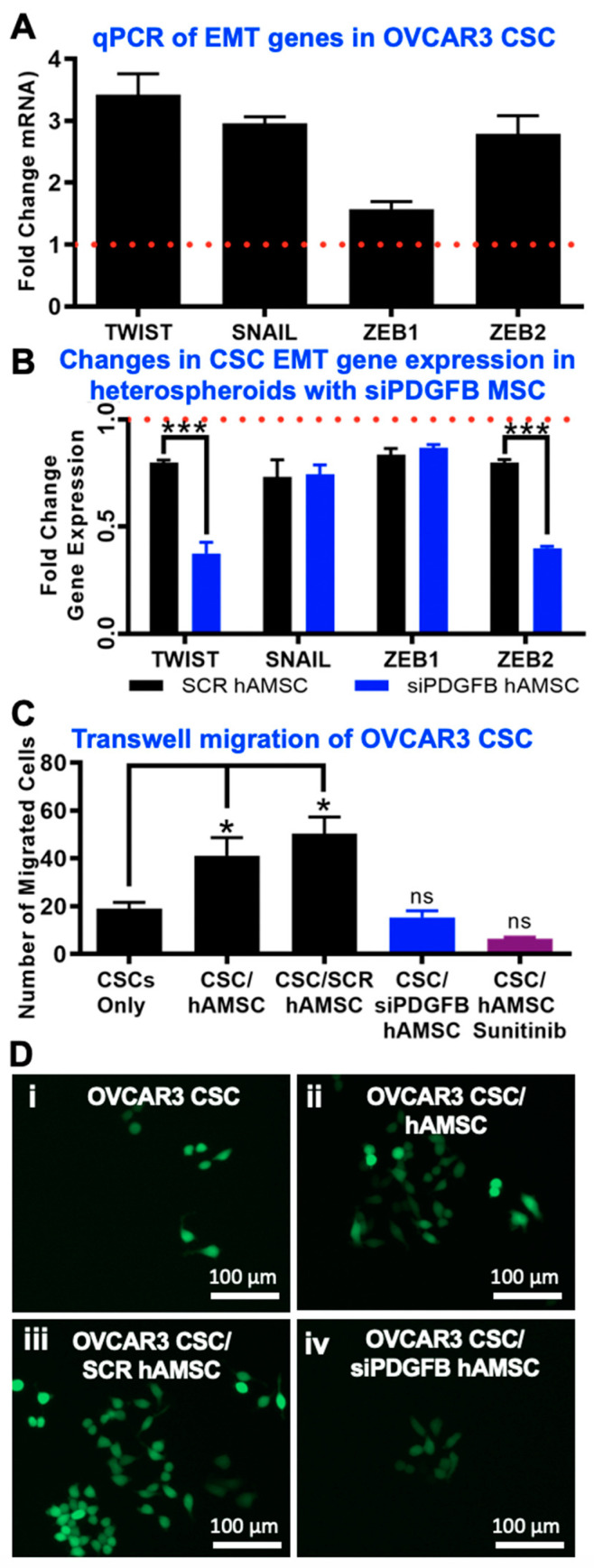
Downstream EMT Markers and Changes in Migration of Ovarian CSC. (**A**) Gene expression analysis of EMT markers in OVCAR3 CSCs cultured with MSCs in heterospheroids, compared to CSC mono-cultures (red dotted line). A significant (>two-fold) increase in gene-expression was observed for several EMT markers tested (*n* ≥ 3). (**B**) Gene expression analysis of EMT markers following CSC/siPDGFB MSC co-cultures demonstrated a significant downregulation (*** *p* < 0.0001, two-way ANOVA, *n* ≥ 3) of EMT markers upon *PDGFB* knockdown. The red line indicates CSC values. (**C**) Increased migration of OVCAR3 CSC was seen in a transwell assay when co-cultured with MSC; this increase was negated with sunitinib treatment or PDGFB knockdown of MSCs (* *p* < 0.05, one-way ANOVA, *n* ≥ 3). (**D**) Representative fluorescent images of migrated GFP+ CSC (green) in a transwell assay after 5 days of co-culture showing (i) OVCAR3 CSCs cultured alone, (ii) OVCAR3 CSC/MSC, (iii) OVCAR3 CSC/SCR MSC and (iv) OVCAR3 CSC/siPDGFB MSC where more migrated cells are seen with MSC culture and siPDGFB reduces the effect MSCs have on CSC migration (*n* ≥ 3). Scale bar = 100 μm.

Increases in EMT transcription markers such as TWIST, SNAIL, ZEB1 and ZEB2 have been linked to increased migration and metastasis, and has previously been linked to PDGF [60,61]. We observed a significant upregulation (>two-fold) of several transcription factors associated with the EMT phenotype in OVCAR3 CSC cultured within CSC/MSC heterospheroids (Figure 5A). The same genes were significantly downregulated in CSC/siPDGFB MSC conditions (*** *p* < 0.0001, two-way ANOVA) (Figure 5B), indicating the partial loss of the EMT phenotype upon loss of PDGF-BB/PDGFR-β signaling. Concurrent with upregulation of EMT genes, we observed that CSC/MSC and CSC/SCR MSC heterospheroids were significantly (* *p* < 0.05, one-way ANOVA) more migratory in a transwell setting, and this migration could be inhibited using the PDGFR-β inhibitor sunitinib or by inhibiting the secretion of PDGF-BB from MSC using siPDGFB MSC within heterospheroids (Figure 5C). Representative fluorescent micrographs are demonstrated in Figure 5D corresponding to the various conditions tested for migration.

### 2.6. Hedgehog Is Activated Downstream of MSC-Derived PDGFB in CSC

**Figure 6:**
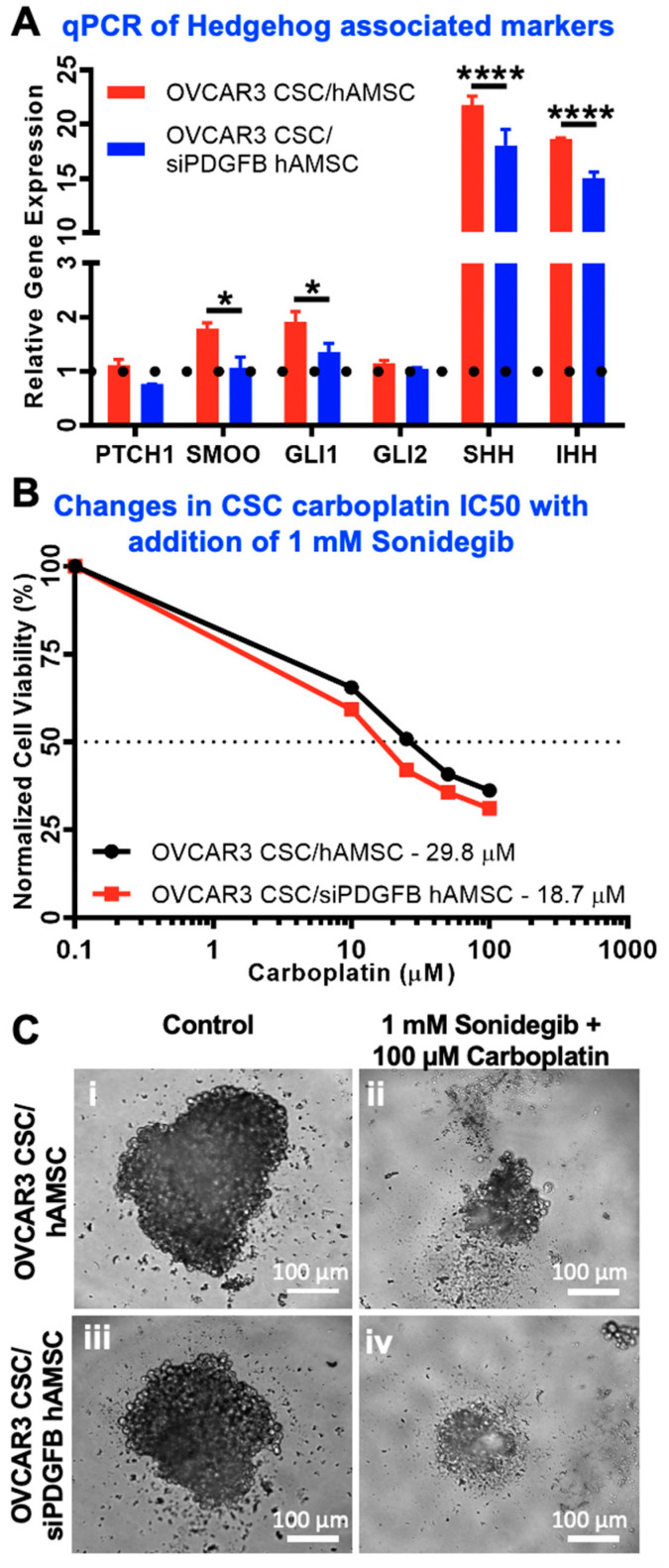
Downstream Activation of Hedgehog through PDGF-BB/PDGFR-β Signaling in Ovarian CSC and MSC Crosstalk. (**A**) Gene expression analysis of Hedgehog pathway elements in OVCAR3 CSC mono-cultures (black line), OVCAR3 CSC/MSC or OVCAR3 CSC/siPDGFB MSC heterospheroids. Gene qPCR data demonstrates a significant loss in Hedgehog ligands (*SHH* and IHH; **** *p* < 0.0001, two-way ANOVA, *n* ≥ 3) upon PDGFB knockdown. Additionally, signaling elements within the Hedgehog pathway are also significantly downregulated (* *p* < 0.05, two-way ANOVA) upon PDGF knockdown including Smoothened (*SMOO*) and *GLI1,* indicating that PDGFB might be upstream of Hedgehog ligand secretion and/or signaling (*n* ≥ 3). (**B**) OVCAR3 CSC/MSC and OVCAR3 CSC/siPDGFB MSC heterospheroids were treated with 1 mM sonidegib, a Smoothened inhibitor, and carboplatin to determine the change in IC_50_. Hedgehog inhibition compounded with knockdown of PDGFB, resulting in a lower carboplatin IC_50_ in CSC/MSC heterospheroids (*n* ≥ 3). (**C**) Representative phase contrast microscopy images of the heterospheroids when treated with 1 mM sonidegib and 100 µM carboplatin, indicating significant visual cell death and loss of spheroid integrity.

We previously showed that MSC induce Hedgehog signaling in CSC [22]. To determine if the PDGF-BB/PDGFR-β signaling was responsible for this alteration in Hedgehog pathway, we used qPCR to observe changes in expression of the genes associated with the Hedgehog signaling pathway (Figure 6A). We found that Hedgehog ligands *SHH* and *IHH* were significantly upregulated in CSC cultured in heterospheroids with WT MSC, and this upregulation was diminished in CSC cultured with siPDGFB MSCs (Figure 6A; **** *p* < 0.0001, two-way ANOVA). Similar patterns of gene expression were observed for downstream targets of classical Hedgehog signaling, *PTCH1, SMO* (* *p* < 0.05, two-way ANOVA) and *GLI1* (* *p* < 0.05, two-way ANOVA), indicating the partial involvement of PDGF-BB signaling upstream of Hedgehog ligand secretion in CSC within the CSC/MSC interaction paradigm. We additionally tested if this cross-talk between PDGF-BB/PDGFR-β and Hedgehog signaling was synergistic by using a SMO inhibitor, sonidegib, at sub-cytotoxic IC_50_ doses of 1 mM. Changes in Carboplatin IC_50_ are plotted in the presence of sonidegib in Figure 6B, implying a significant cross-talk between the two tested pathways. Representative phase contrast images of spheroids treated with carboplatin in the presence of sonidegib are shown in Figure 6C. Table S1 lists the carboplatin IC_50_ across all comparison groups, with and without sonidegib.

### 2.7. Serial Passaging of CSC/MSC Spheroids Indicate CSC Enrichment

**Figure 7:**
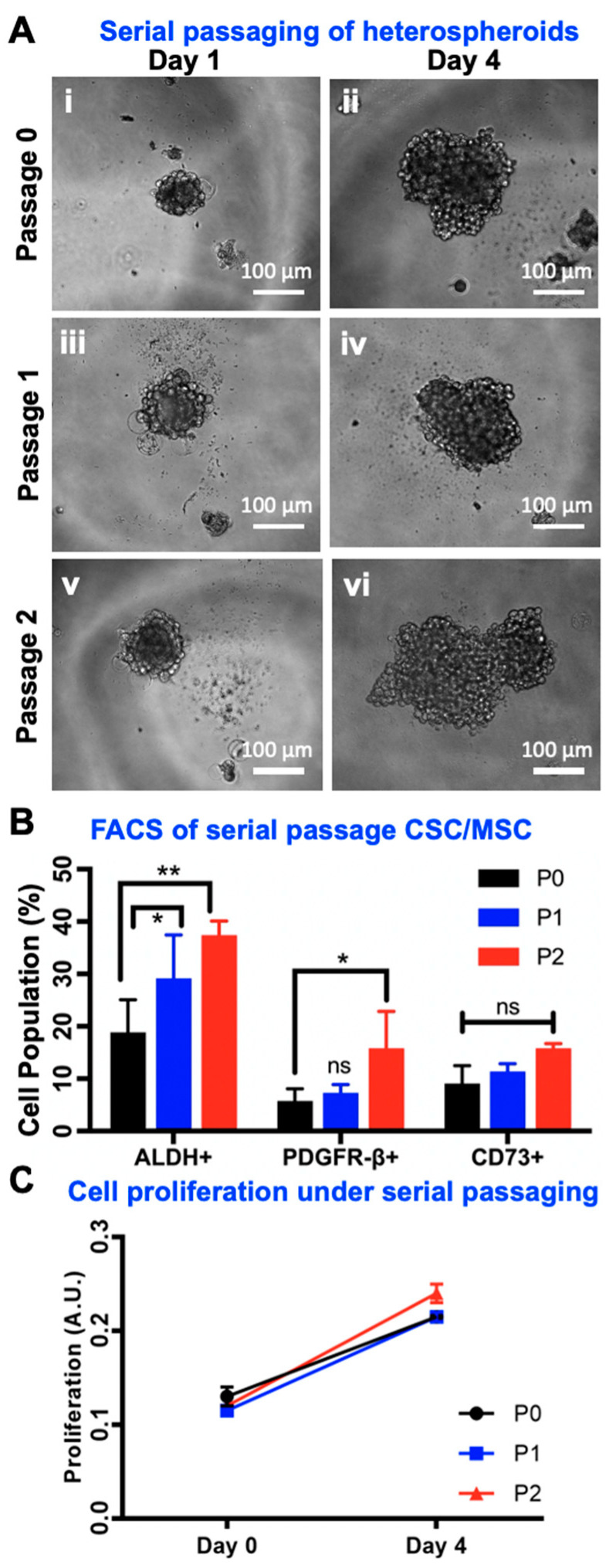
MSC Support the Enrichment of CSC due to Serial Passaging. (**A**) Representative optical microscopy images of CSC/MSC heterospheroids on Day 1 and Day 4, Passage 0–2. (**B**) Quantification of the change in ALDH+, PDGFR-β and CD73+ cell populations with FACS in each passage. An increase over passage number is seen in ALDH and PDGFR-β but the CD73 population remains constant (* *p* < 0.05, ** *p* < 0.001, two-way ANOVA, *n* ≥ 3). (**C**) Proliferation quantified on Day 0 and Day 4 was consistent across three passages (paired *t*-test, *n* ≥ 3).

We have recently demonstrated that serially passaging ovarian cancer spheroids over 20 passages increases their stemness, tumorigenicity and chemoresistance [21]. In order to observe any changes in CSC enrichment modulated by co-culturing with MSC in heterospheroids, we serially passaged CSC/MSC spheroids for three consecutive passages (Passage 0–2). Three-dimensional serial passaging did not diminish the ability of CSC and MSC to form spheroids, as demonstrated by phase contrast images of spheroids at Day 1 and Day 4 (Figure 7A). Flow analysis at each passage indicated the statistically significant increase in ALDH+ cells within heterospheroids with serial passaging (** *p* < 0.001, two-way ANOVA), similar to our previous serial passage work [47], and a significant increase in PDGFR-β expression (* *p* < 0.05, two-way ANOVA). No changes in CD73 expression were noted, implying the steady maintenance of ~20% of the heterospheroid population as MSC (Figure 7B). Furthermore, no significant changes in proliferation were observed in heterospheroids with serial passaging (Figure 7C).

## 3. Discussion

Ovarian CSC are implicated in platinum resistance, disease recurrence and metastasis, leading to increased tumorigenicity and disease relapse. CSC harness the power of the tumor microenvironment (TME), including carcinoma associated mesenchymal stem cells (CA-MSC), among other stromal cells, to achieve their tumorigenic goals. The presence of CA-MSC contribute specifically to platinum resistance via multiple documented signaling pathways and mechanisms [22,29,62]. Well-established cytokine/chemokine networks exist to indicate how CA-MSC communicate with CSC and promote tumorigenesis and metastasis [11,12,13].

Here, we explored a novel link between the two cell types in the ovarian cancer TME, previously unknown in its involvement in CSC/CA-MSC communication, namely the PDGF-BB/PDGFR-β signaling pathway. De-regulation of PDGF-BB/PDGFR-β signaling is implicated in driving tumorigenesis and metastasis in several cancers [63,64,65]. PDGF-BB, the principal ligand for the PDGFR-β receptor, is significantly elevated in malignant ascites compared to non-malignant peritoneal fluid. In fact, targeting the PDGF receptors has been shown to inhibit PDGF-induced receptor activation and cell proliferation in ovarian cancer cells [66].

We found PDGFR-β receptor expression is upregulated in CSC co-cultured with MSC, likely in response to paracrine stimulation from PDGF-BB secretion by MSC. In the context of platinum resistance, our results clearly demonstrate that removing PDGF-BB from the shared CSC/MSC microenvironment through gene knockdown or inhibiting PDGF-BB/PDGFR-β interactions through sunitinib significantly improves sensitivity to carboplatin. This is generally in line with evidence in colorectal and other carcinomas where CA-MSC increase platinum resistance through a variety of mechanisms [22,29,62]. Our work demonstrates the specific involvement of the growth factor PDGF-BB in this platinum resistance phenomenon, in line with other described roles of PDGF signaling in ovarian and other cancers in their interaction with mesenchymal stroma [36,37,67]. Furthermore, in line with established literature, we observe an increase in the percentage of ALDH^+^ CSC in the heterospheroids, with an associated increase in EMT signatures, and functional migration [40,41,43,44,46,47,66]. PDGF signaling has been related to increased EMT markers such as *TWIST, SNAIL, ZEB1* and *ZEB2* through several pathways in cancer cell-MSC interactions [44,47,68,69]. 

**Figure 8:**
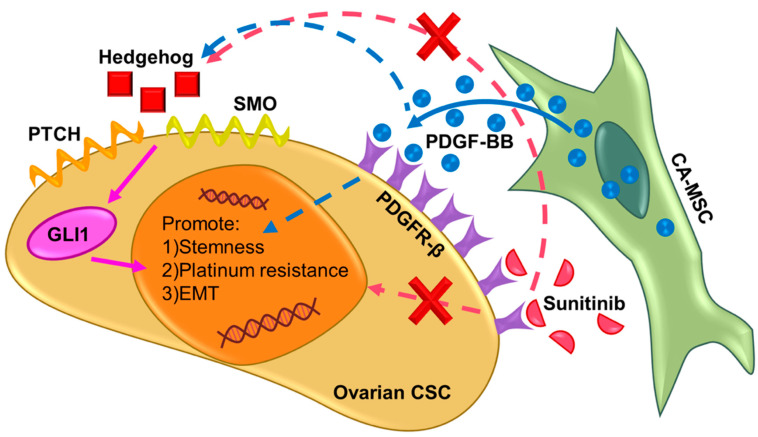
Hypothesized mechanism of PDGF-BB/PDGFR-β communication between ovarian CSC and CA-MSC with Hedgehog downstream signaling. PDGF-BB is secreted by CA-MSC and binds to PDGFR-β receptor, which are expressed by ovarian CSC. Binding between PDGFR-β and PDGF-BB directly causes an increase in CSC markers, platinum resistance and EMT markers in ovarian CSC. Additionally, PDGF-BB binding is shown to be upstream of Hedgehog as it leads to an increase in Hedgehog ligand secretion. Hedgehog is known to also cause an increase in CSC markers, platinum resistance and EMT markers in ovarian CSC through PTCH, SMO and GLI1. Treatment with sunitinib, a PDGFR-β inhibitor, leads to decrease in the CSC and EMT marker expression, and increase in sensitivity to platinum. These phenotypes are direct result of inhibition of the PDGF-BB/PDGFR-β and Hedgehog signaling pathways.

The Hedgehog signaling pathway has been well-documented to promote platinum resistance and tumorigenesis of epithelial ovarian tumors, even posing as an attractive therapeutic target [70,71]. Our results indicate that CSC/MSC interactions increase the secretion of Hedgehog ligands, consistent with the bi-directional communication paradigm resulting in increased percentage of ALDH^+^ CSC via MSC-secreted BMP-4 [9]. Our studies further reveal the putative upstream involvement of PDGF-BB/PDGFR-β potentiating CSC/MSC Hedgehog signaling through *PTCH, SMO* and *GLI1*, all documented to result in platinum resistance of ovarian cancer [6,22,45,72]. Knockdown of MSC-derived PDGF-BB results in a significant loss of Hedgehog ligands (SHH, IHH) and pathway elements (PTCH, SMO, GLI1) in the CSC compartment, wholly implicating PDGF-BB to be a potent upstream regulator of Hedgehog signaling. Paracrine PDGF-BB is implicated upstream of Hedgehog, resulting in migration and cytoprotection in non-cancerous muscle cells, and several cancers including gliomas and cholangiocarcinomas [45,73,74,75]. Through our results, we therefore propose a novel cross-talk between PDGF-BB/PDGFR-β interactions and Hedgehog signaling in ovarian cancer, which can lead to the development of a tumorigenic, metastatic and platinum resistant phenotype within CSC, driven partially by MSC-secreted PDGF-BB (Figure 8).

## 4. Materials and Methods

### 4.1. Materials

All tissue culture reagents (medium components, supplements) were purchased from Thermo Fisher Scientific (Waltham, MA, USA), unless otherwise specified. The OVCAR3 cell line was purchased from American Type Culture Collection (ATCC) (Waltham, MA, USA), and used under Passage 40. Human adipose-derived mesenchymal cells were also purchased from Thermo Fisher Scientific, and used within Passage 5. RNA interference reagents were purchased from Millipore Sigma (St. Louis, MO, USA). Matched primary dissociated live cryopreserved patient samples from high grade serous ovarian carcinoma were consented to Memorial Sloan Kettering Cancer Center IRB-approved biospecimen banking protocol and analyzed under an IRB-approved biospecimen use protocol. Sonidegib was a gift from the laboratory of Dr. Charlotte Mistretta at the University of Michigan School of Dentistry (Ann Arbor, MI, USA). The study was approved by the Memorial Sloan Kettering Cancer Center Institutional Review Board (protocol 15-200, initial approval 8/21/2015, continuing review approval 7/23/2019).

### 4.2. Isolation of Ovarian CSCs and CA-MSCs from Ovarian Cancer Patient Samples

CSC were isolated from the OVCAR3 cell line, or two high-grade serous ovarian carcinomas (Patient 1 and Patient 2), using protocols outlined previously [19,55,56,57,58]. Briefly, single cell suspensions were incubated with ALDEFLUOR reagent (Stem Cell Technologies, Vancouver, BC, Canada), and CD133 antibody. In this work, we define the ‘ALDH+ cells’ as the ‘viable cells that possess high ALDH activity in the ALDEFLOUR FACS assay.’ ALDH+/CD133+ cells were isolated using fluorescent activated cell sorting on the Beckman Coulter MoFlo Astrios, by trained technicians.

For CA-MSC from single cell tumor suspensions, cells were plated onto two-dimensional (2D) tissue culture treated plates in an MSC selection medium following protocols outlined previously [28]. The medium consisted of basal MEBM (Lonza, Walkesville, MD, USA), supplemented with 20 ng/mL EGF, bFGF, 1× B27, 5 µg/mL Insulin, 100 µM β-mercaptoethanol, 1 ng/mL hydrocortisone, 1× antibiotics/antimycotics and 10% fetal bovine serum. Adherent cells were CA-MSC. 

### 4.3. RNA Interference of PDGFB in MSC

Validated siRNA duplex constructs targeted against human *PDGFB* were purchased from Sigma Aldrich (SASI_Hs01_00121961, SASI_Hs01_00121962, (St. Louis, MO, USA). Mission siRNA Transfection reagent (Sigma Aldrich) was used to reverse transfect 10 nM of siRNA to MSC, in a 24-well format following manufacturer’s protocols. A universal negative siRNA non-targeting control (Sigma Aldrich) was utilized to rule out effects from transfection protocols. RNA was extracted from MSC treated with siRNA (siPDGFB MSC) to assess the amount of knockdown via qPCR, outlined below. 

### 4.4. Formation of Mono- and Heterospheroids from CSC and MSC

Mono- or heterospheroids were generated on an in vitro 384 well hanging drop array platform as described previously [19,55,56,57,58]. Briefly, to generate CSC monospheroids, single cell suspensions of CSC were diluted, in such a manner that a 20 µL drop contained 12 CSCs. For heterospheroids, CSC and MSC were diluted in a 1:3 ratio, such that a 20 µL drop contained three CSCs and nine MSCs. Spheroids were cultured in a 1:1 medium mixture of RPMI and ADSC-BM (Lonza) supplemented with 1% penicillin/streptomycin and 1% L-glutamate. Live cell imaging using phase contrast microscopy was utilized to follow the formation of CSC or CSC/MSC spheroids. Spheroids were generated from CSCs isolated from the OVCAR3 cell line, or OVCAR3 CSCs with human adipose MSC (MSC). Two matched patient samples (Patient 1 and Patient 2) were also utilized to generate CSC/CA-MSC spheroids.

Additionally, heterospheroids were manufactured from CSC and siPDGFB MSC, in order to assess changes in platinum resistance and CSC characteristics within heterospheroids. CSC^gfp^/siPDGFB MSC heterospheroids were also generated, to be able to isolate the CSC^gfp^ compartment using fluorescent activated cell sorting using protocols described previously [20], for subsequent qPCR analysis.

### 4.5. Flow Cytometry Analysis

At the end of 4 days in spheroid culture, mono- or heterospheroids were harvested and resuspended in FACS Buffer (PBS + 2% fetal bovine serum). The presence of MSC was determined by staining with CD73. CSC were identified using elevated ALDH activity using the ALDEFLUOR Kit. The presence of PDGFR-β (CD340) was assessed by staining with PDGFR-β antibody. Flow analysis protocols were adapted from our previous studies [19,20,21,55,56], and gating strategies are demonstrated in Supplementary Information Figures S1–S4.

### 4.6. Quantification of Cytokines Using ELISA

For ELISA assays, medium was harvested from 100 spheroids (CSC, CSC/CA-MSC spheroids). ELISA assays were performed using the Duoset ELISA system (R&D Biosystems, Minneapolis MN) following the manufacturer’s protocol, modified to include an overnight incubation. PDGF-BB was analyzed using a standard curve, and a four parametric ELISA curve generation. ELISA assays and data analysis were performed at the Immunological Monitoring Core at the Rogel Cancer Center, University of Michigan (Ann Arbor, MI, USA).

### 4.7. Assessment of Chemoresistance in Spheroids

Spheroids were generated from CSC, CSC/MSC or CSC/siPDGFB MSC and allowed to form until Day 5. To identify changes in IC_50_ for carboplatin, spheroids were treated with a range of concentrations of Carboplatin (10–200 µM) for 48 h. The MTS viability assay (Abcam, Cambridge, UK) was used to determine viability of control untreated and carboplatin-treated spheroids, following manufacturer’s protocols. Following a 1.5-h incubation of the MTS reagent with spheroids at 37 °C, absorbance was read at 590 nm following manufacturer’s protocols. Results were quantified as normalized cell viability, based on the viability of untreated control spheroids. Experiments were repeated with three–five biological replicates for statistical analysis, and each experiment contained >20 technical replicates. For combination drug doses, a range of carboplatin was utilized along with Sunitinib 5 µM, or Sonidegib 1 mM, to identify changes in IC_50_.

### 4.8. Assessment of Migration in Spheroids

In order to quantify invasiveness of heterospheroids, 8 µm transwell inserts were placed in each well of a 24-well plate. Ten CSC^gfp^ or CSC^gfp^/MSC spheroids were harvested at Day 5 from hanging drop arrays, and a single cell suspension was made. The cells were then FACS-sorted for the GFP expression [20]. GFP-positive single cells were placed on the top chamber of a transwell insert. The bottom chamber was filled with 400 µL of fresh medium, thus, only the bottom of the transwell insert was immersed in medium. Following 3 days, the transwell insert was removed, and several images of the bottom of the 24-well plate were obtained using fluorescent microscopy, identifying GFP+ cells. Image J (U.S. National Institutes of Health, Bethesda, MD, USA) was used to quantify the number of cells in a field of view. At least four random non-overlapping fields of view were counted from each experiment, to find the number of cells that migrated through the transwell insert to the bottom of the well.

### 4.9. Gene Expression Analysis via qPCR

RNA was extracted from spheroids or cells using the RNeasy extraction kit (Qiagen, Hilden, Germany). Extracted RNA was assessed for concentration and purity using a Nanodrop 2000 (Thermo Fisher Scientific) spectrophotometer. RNA was transcribed to cDNA using the High-fidelity cDNA Transcription kit (Life Technologies, Carlsbad, CA, USA), and qPCR was carried out in a 96-well format using 7900HT (Applied Biosystems, Foster City, CA, USA). Gene expression differences were quantified using the 2ΔΔC_T_ method, using GAPDH as the housekeeping control, and reported as fold change compared to a control sample. The following primers were utilized to test changes in EMT: *TWIST, SNAIL, ZEB1, ZEB2*; the following primers were utilized to test PDGF signaling: *PDGFB, PDGFRB*; the following primers were used to test Hedgehog signaling: *PTCH1, SMOO, GLI1, GLI2, SHH, IHH*. A list of all primers used in qPCR is provided in Table S2.

For qPCR experiments, RNA was isolated from CSC mono-spheroids, or CSCs isolated based on a GFP label from CSC^gfp^/MSC heterospheroids or CSC^gfp^/siPDGFB MSC spheroids. Control comparisons were made with CSC mono-spheroids using the 2ΔΔC_T_ method, and fold changes are reported.

### 4.10. Serial Passaging of CSC/MSC Heterospheroids

In order to assess CSC enrichment, and predict tumorigenicity of CSC/MSC heterospheroids, we utilized an in vitro serial passage model [20,21]. Passage 0 (P0) spheroids were generated using protocols outlined in Section 4.4. Following 4–5 days of heterospheroid culture, spheroids were harvested and CSC were isolated based on elevated ALDH activity using fluorescent activated cell sorting. Isolated CSC were combined with MSC again and seeded into heterospheroids, and allowed to remain in non-adherent hanging drop culture for 4–5 days. This process was repeated four times, until a terminal passage of P3 was reached. At the end of each passage, spheroids were also harvested to analyze the expression of ALDH activity, and PDGFR-β and the maintenance of a CD73+ MSC population, using flow cytometry. Proliferation was assessed in spheroids by comparing MTS absorbance in Day 1 compared to Day 4.

### 4.11. Data Analysis and Statistics

All experiments were performed with three–five biological replicates, with at least 20 technical replicates for drug viability and at least three technical replicates for qPCR. Statistical analysis was performed using GraphPad Prism 8 for Mac Os X (www.graphpad.com). Appropriate statistical tests were performed and reported in each graph/figure legend, with asterisks indicating any significant differences in test variables.

## 5. Conclusions

In conclusion, we have found PDGF-BB/PDGF-β to be an important signaling pathway between CA-MSC and CSC in ovarian cancer, which has yet to be fully studied. We have linked PDGF-BB/PDGF-β with increased stemness, metastatic potential and platinum resistance in ovarian CSC, acting in part via canonical Hedgehog signaling. Inhibition of PDGF-BB/PDGF-β signaling was found to reduce platinum resistance and metastatic potential development in ovarian CSC. Our findings suggest that this PDGF-BB/PDGF-β pathway holds much interest for future studies in designing more effective therapies for ovarian cancer. Overall, our report underscores the importance of TME signaling, and suggests that in order to effectively target ovarian cancers, the tumor cells, as well as the supporting stromal cells, are important, and need to be considered together.

## Supplementary Materials

The following are available online at https://www.mdpi.com/2072-6694/12/8/2063/s1, Figure S1: FACS gating strategy for CD73 expression in OVCAR3 and patient-derived CSC. FMO isotype for CD73 expression in (A) OVCAR3 CSC, (B) Patient 1 CSC and (C) Patient 2 CSC, Figure S2: FACS gating strategy for MSC expression of CD73, CD105 and ALDH, Figure S3: FACS gating strategy for siPDGFB-MSC CD73 expression, Figure S4: FACS gating strategy for PDGF-β and ALDH in patient CSC, Table S1: Summary of Carboplatin IC_50_ Values in OVCAR3/MSC heterospheroids, Table S2: Primers used in qPCR.
