# Supplementary material for: Carcinoma-Associated Mesenchymal Stem Cells Promote Chemoresistance in Ovarian Cancer Stem Cells via PDGF Signaling
Source: Cancers (Basel). 2020 Jul 27;12(8):2063. doi: 10.3390/cancers12082063 (PMC7464970; doi:10.3390/cancers12082063)
Supplement: Supplementary file 1 [file cancers-12-02063-s001.pdf]

cancers

MDPI

Article

Carcinoma-Associated Mesenchymal Stem Cells Promote Chemoresistance in Ovarian Cancer Stem Cells via PDGF Signaling

Shreya Raghavan, Catherine Snyder, Anni Wang, Karen McLean, Dmitriy Zamarin, Ronald J. Buckanovich and Geeta Mehta

Supplementary Materials

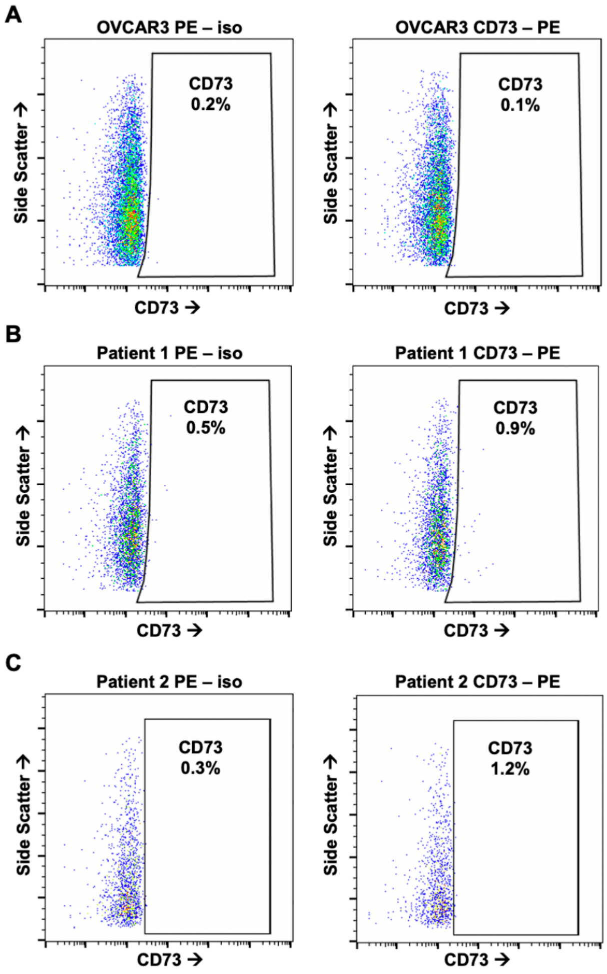

Supplementary Figure S1. FACS gating strategy for CD73 expression in OVCAR3 and patient-derived CSC. (Left) FMO isotype for CD73 expression in (**A**) OVCAR3 CSC, (**B**) Patient 1 CSC, and (**C**) Patient 2 CSC. (Right) FACS of CD73 expression in (**A**) OVCAR3 CSC, (**B**) Patient 1 CSC, and (**C**) Patient 2 CSC showing that any CD73 signal from CSC/MSC heterospheroids is from CD73 expressing MSC.

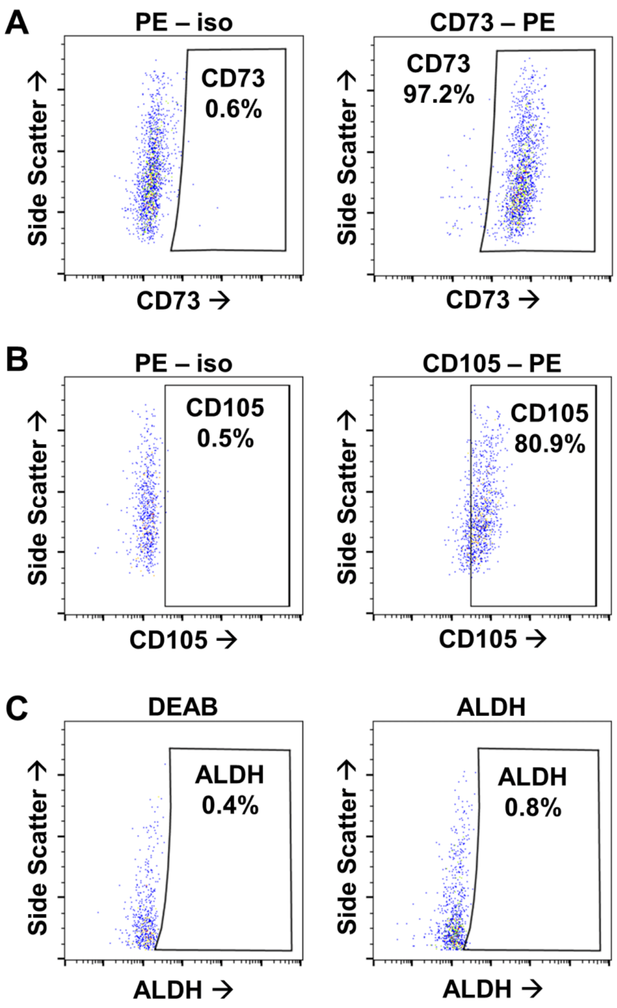

Supplementary Figure S2. FACS gating strategy for MSC expression of CD73, CD105 and ALDH. (**A**) Gating strategy for CD73 using a PE isotype. (**B**) Gating strategy for CD105 with a PE isotype. (**C**) Gating strategy for ALDH using DEAB.

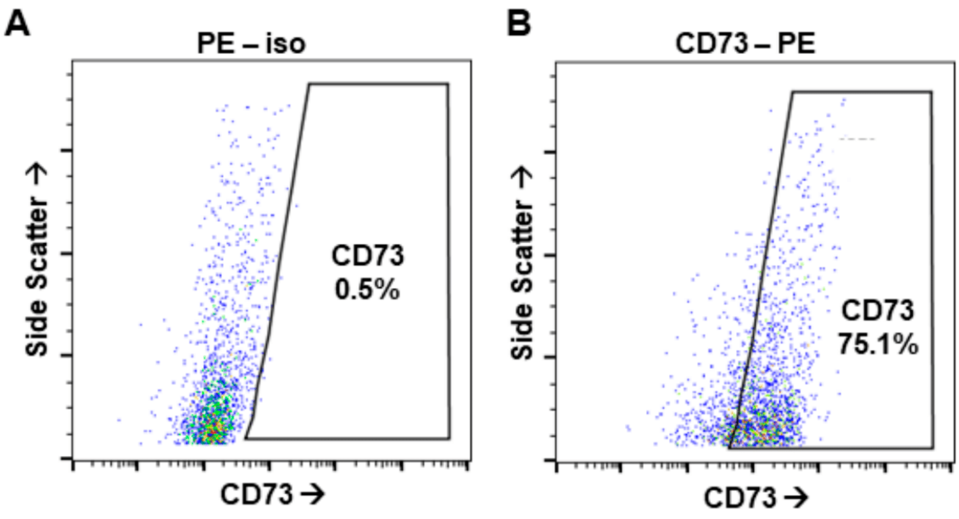

Supplementary Figure S3. FACS gating strategy for siPDGFB-MSC CD73 expression. (**A**) Gating strategy for CD73 using a PE isotype. (**B**) FACS of CD73 expression showing that MSC maintain a mesenchymal phenotype even with PDGFB silencing.

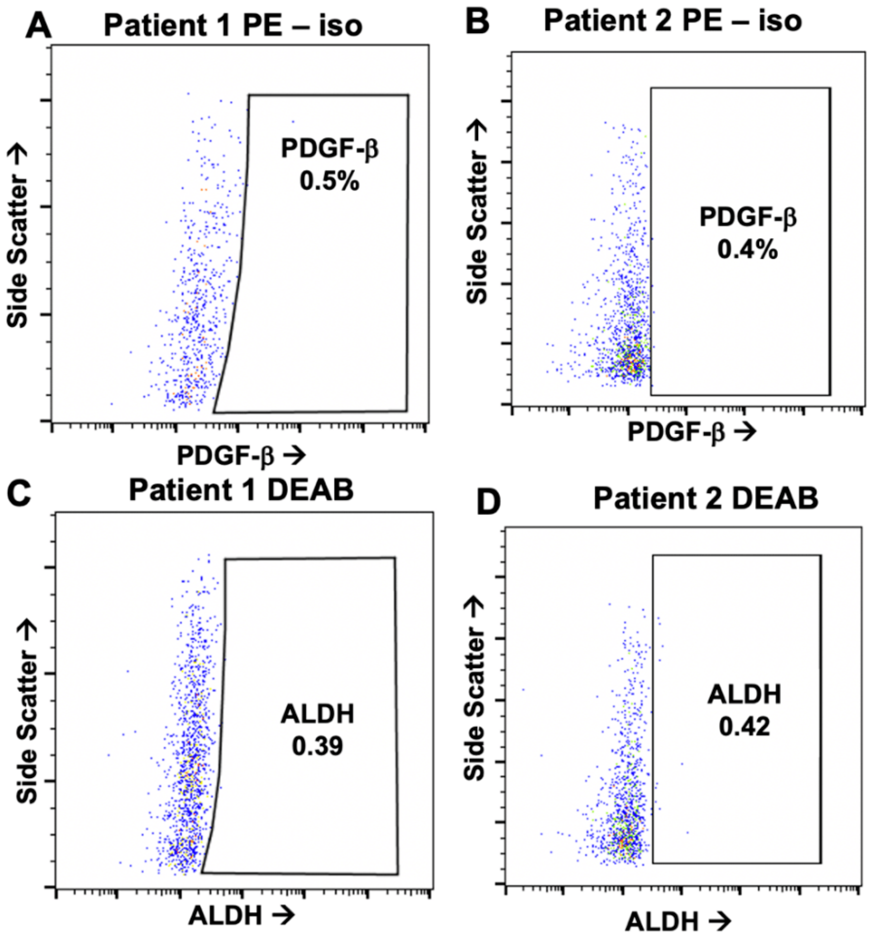

Supplementary Figure S4. FACS gating strategy for PDGF-b and ALDH in patient derived CSC. (**A**) and (**B**) Gating strategy for PDGF-b using a PE isotype. (**C**) and (**D**) Gating strategy for ALDH expression using DEAB.

Table S1. Summary of Carboplatin IC50 Values in OVCAR3/MSC Heterospheroids

Condition	Carboplatin IC_{50} (μM)	Manuscript Figure
OVCAR3 CSC	25.7	2D
OVCAR3 CSC/hAMSC	67.0	2D
OVCAR3 CSC + Sunitinib	27.3	3E
OVCAR3 CSC/hAMSC + Sunitinib	39.4	3E
OVCAR3 CSC/SCR hAMSC	66.3	4D
OVCAR3 CSC/siPDGFB hAMSC	46.2	4D
OVCAR3 CSC/hAMSC + Sonidegib	29.8	6B
OVCAR3 CSC/siPDGFB hAMSC + Sonidegib	18.7	6B

Table S2. Primers used in qPCR.

Role	Primers	Sequence
CSC EMT	TWIST	5′- GTC CGC AGT CTT ACG AGG AG -3′<br>5′- GCT TGA GGG TCT GAA TCT TGC T -3′
	SNAIL	5′- TCG GAA GCC TAA CTA CAG CGA -3′<br>5′- AGA TGA GCA TTG GCA GCG AG -3′
	ZEB1	5′- GAT GAT GAA TGC GAG TCA GAT GC -3′<br>5′- ACA GCA GTG TCT TGT TGT TGT -3′
	ZEB2	5′- GGA GAC GAG TCC AGC TAG TGT -3′<br>5′- CCA CTC CAC CCT CCC TTA TTT C -3′
PDGF Signaling	PDGFB	5′- CTC GAT CCG CTC CTT TGA TGA -3′<br>5′- CGT TGG TGC GGT CTA TGA G -3′
	PDGFRB	5′- TGA TGC CGA GGA ACT ATT CAT CT -3′<br>5′- TTT CTT CTC GTG CAG TGT CAC -3′
Hedgehog Signaling	Patch 1 (PTCH1)	5′- ACT TCA AGG GGT ACG AGT ATG T -3′<br>5′- TGC GAC ACT CTG ATG AAC CAC -3′
	Smoothened (SMOO)	5′- TCG AAT CGC TAC CCT GCT G -3′<br>5′- CAA GCC TCA TGG TGC CAT CT -3′
	GLI1	5′- AAC GCT ATA CAG ATC CTA GCT CG -3′<br>5′- GTG CCG TTT GGT CAC ATG G -3′
	GLI2	5′- CCC CTA CCG ATT GAC ATG CG -3′<br>5′- GAA AGC CGG ATC AAG GAG ATG -3′
	Sonic Hedgehog (SHH)	5′- CTC GCT GCT GGT ATG CTC G -3′<br>5′- ATC GCT CGG AGT TTC TGG AGA -3′
	Indian Hedgehog (IHH)	5′- AGA CCG CGA CCG CAA TAA G -3′<br>5′- GCC TTT GAC TCG TAA TAC ACC CA -3′

© 2020 by the authors. Licensee MDPI, Basel, Switzerland. This article is an open access article distributed under the terms and conditions of the Creative Commons Attribution (CC BY) license (http://creativecommons.org/licenses/by/4.0/).
